# Proteomic Analysis of Membrane Blebs of *Brucella abortus* 2308 and RB51 and Their Evaluation as an Acellular Vaccine

**DOI:** 10.3389/fmicb.2019.02714

**Published:** 2019-11-29

**Authors:** Minerva Araiza-Villanueva, Eric Daniel Avila-Calderón, Leopoldo Flores-Romo, Juana Calderón-Amador, Nammalwar Sriranganathan, Hamzeh Al Qublan, Sharon Witonsky, Ma. Guadalupe Aguilera-Arreola, María del Socorro Ruiz-Palma, Enrico A. Ruiz, Francisco Suárez-Güemes, Zulema Gómez-Lunar, Araceli Contreras-Rodríguez

**Affiliations:** ^1^Departamento de Microbiología, Escuela Nacional de Ciencias Biológicas, Instituto Politécnico Nacional, Mexico City, Mexico; ^2^Departamento de Biología Celular, Centro de Investigación y de Estudios Avanzados, Instituto Politécnico Nacional, CINVESTAV-IPN, Mexico City, Mexico; ^3^Center for One Health Research, Virginia-Maryland College of Veterinary Medicine, Virginia Tech, Blacksburg, VA, United States; ^4^Large Animal Clinical Sciences, Virginia-Maryland College of Veterinary Medicine, Virginia Tech, Blacksburg, VA, United States; ^5^División Químico-Biológicas, Universidad Tecnológica de Tecámac, Tecámac, Mexico; ^6^Departamento de Zoología, Escuela Nacional de Ciencias Biológicas, Instituto Politécnico Nacional, Mexico City, Mexico; ^7^Departamento de Microbiología e Inmunología, Facultad de Medicina Veterinaria y Zootecnia, Universidad Nacional Autónoma de México, Mexico City, Mexico

**Keywords:** membrane blebs, *Brucella abortus*, vaccines, Gram-negative bacteria, brucellosis

## Abstract

Membrane blebs are released from Gram-negative bacteria, however, little is known about *Brucella* blebs. This work pursued two objectives, the first was to determine and identify the proteins in the membrane blebs by proteomics and *in silico* analysis. The second aim was to evaluate the use of membrane blebs of *Brucella abortus* 2308 and *B. abortus* RB51 as an acellular vaccine *in vivo* and *in vitro*. To achieve these aims, membrane blebs from *B. abortus* 2308 and RB51 were obtained and then analyzed by liquid chromatography coupled to mass spectrometry. *Brucella* membrane blebs were used as a “vaccine” to induce an immune response in BALB/c mice, using the strain *B. abortus* RB51 as a positive vaccine control. After subsequent challenge with *B. abortus* 2308, CFUs in spleens were determined; and immunoglobulins IgG1 and IgG2a were measured in murine serum by ELISA. Also, activation and costimulatory molecules induced by membrane blebs were analyzed in splenocytes by flow cytometry. Two hundred and twenty eight proteins were identified in 2308 membrane blebs and 171 in RB51 blebs, some of them are well-known *Brucella* immunogens such as SodC, Omp2b, Omp2a, Omp10, Omp16, and Omp19. Mice immunized with membrane blebs from rough or smooth *B. abortus* induced similar protective immune responses as well as the vaccine *B. abortus* RB51 after the challenge with virulent strain *B. abortus* 2308 (*P* < 0.05). The levels of IgG2a in mice vaccinated with 2308 membrane blebs were higher than those vaccinated with RB51 membrane blebs or *B. abortus* RB51 post-boosting. Moreover, mice immunized with 2308 blebs increased the percentage of activated B cells (CD19^+^CD69^+^) *in vitro.* Therefore, membrane blebs are potential candidates for the development of an acellular vaccine against brucellosis, especially those derived from the rough strains so that serological diagnostic is not affected.

## Introduction

The membrane blebs have been described in Gram-negative bacteria for more than 50 years (Manning and Kuehn, 2013). These membrane blebs are released from the outer membrane of the cell to the external milieu, showing spherical shapes, and ranging in sizes from 20 to 250 nm. In general, membrane blebs are composed of outer membrane proteins (OMPs), periplasmic and cytoplasmic proteins, phospholipids, lipopolysaccharide (LPS), as well as DNA and RNA (Bitto et al., 2017; Jan, 2017). Several authors have described the release of membrane blebs from different pathogens, as well as their effects *in vitro* or *in vivo* (Holst et al., 2009; McConnell et al., 2011; Stevenson et al., 2018; Zhang et al., 2018). Recently, Marion et al. (2019), showed pulmonary inflammation and neutrophil recruitment as well as cytokine production in mice inoculated with *Acinetobacter baumannii* membrane blebs intranasally. Also, membrane blebs from *Aeromonas hydrophila* induced B and T cell activation and the production of TNFα, IL-1β, and stimulated peripheral blood mononuclear cells producing IL-8 (Avila-Calderón et al., 2018).

Membrane blebs have been involved in protein transportation including virulence factors, nutrient acquisition, horizontal genetic transfer, bactericidal action, inter-kingdom communication, and modulation of the host immune response (Jan, 2017; Lynch and Alegado, 2017; Yu et al., 2017).

Because of the amount of antigen present in membrane blebs and its ability to modulate the immune response, they have been tested as acellular vaccines candidates (Cai et al., 2018). Membrane blebs from different pathogens such as *Escherichia coli, Vibrio cholerae or Neisseria meningitidis* have been used to immunize animals and then challenge them with the progenitor pathogenic bacterium to assess the efficacy of the protective immune response (Yu et al., 2017; Cai et al., 2018). One of the characteristics that make membrane blebs attractive to develop vaccines is that these contain natural components of the bacterial cells, so they may induce an immune response *in vitro* and *in vivo* but without generating infection in the host, because blebs are considering acellular entities. Membrane blebs also have shown to be good adjuvants (Tan et al., 2018). For example, blebs from *Neisseria meningitidis* mixed with capsular polysaccharide from meningococcal A group were used as adjuvants to immunize rabbits, increasing the humoral immune response (Siadat et al., 2011). Because membrane blebs both contain cellular components, such as antigenic proteins, but they are not virulent, as they aren’t the whole cell, both of these characteristics make them safer, secure and effective platform to develop acellular vaccines.

The vaccines currently used against animal brucellosis are based on live attenuated cells. The most widely used vaccines in the world to protect cattle against brucellosis are *B. abortus* strain 19 (S19) and RB51; the first one is a smooth strain, whereas RB51 is a rough mutant (Schurig et al., 1991; Avila-Calderón et al., 2013). All commercially available vaccines including those based on *B. abortus* strains are virulent for humans. Some occupational human infections have been reported, as well as infection due to raw milk consumption since vaccine strains have been shown to be excreted for short periods of time in the milk (Wallach et al., 2008; Cossaboom et al., 2018). Therefore, it is important to develop a safer acellular vaccine against human brucellosis.

Membrane blebs from *B. melitensis* 16M and the rough mutant VTRM1 induced protection in mice against the challenge with virulent *B. melitensis*. The level of protection was similar to that achieved by the live vaccine *B. melitensis* Rev 1. The composition of *B. melitensis* membrane blebs revealed proteins involved in the immune response (Avila-Calderón et al., 2012). Because *Brucella* is an intracellular pathogen, it is important that the vaccine elicits a cellular immune response to provide protection. Several subunit vaccines based on purified proteins have also been tested, however, in most of the cases, these proteins provided moderate levels of protection compared with live vaccine control (Avila-Calderón et al., 2013).

The aims of this work, were to identify the protein cargo of membrane blebs and to analyze the protective immune response induced by blebs from *B. abortus* 2308 and RB51 in the murine model. To reach these aims, the protein cargo in membrane blebs from both strains were performed through a proteomic and *in silico* analysis. Membrane blebs obtained from *B. abortus* 2308 and *B. abortus* RB51 were used to immunize mice and then challenged with virulent *B. abortus* 2308, the residual bacterial burden in spleen, the induction of IgG subtype antibodies as well as the expression of surface activation markers on lymphocytes and antigen presenting cells (APCs) were determined.

## Materials and Methods

### Bacterial Strains

The smooth strain *B. abortus* 2308, *B. abortus* RB51 a naturally occurring rough strain which is used as a vaccine, and the Group B *Salmonella* sp. were used. *Brucella* strains were cultured on tryptic soy agar supplemented with 0.5% yeast extract (TSA-YE), whereas *Salmonella* strain was cultured on tryptic soy agar (TSA) plates. Prior to obtaining the membrane blebs, antigenic variation from both strains was checked by agglutination by agglutination with polyclonal anti-*Brucella* serum (MICSA^®^) and acriflavine 0.1%.

### Obtaining Blebs of *B. abortus*

The membrane blebs were obtained following the protocol described previously (Avila-Calderón et al., 2012; Cooke et al., 2019; Bagheri-Nejad et al., 2019). Briefly, *B. abortus* strain 2308 and the vaccine *B. abortus* strain RB51 were cultured in large-scale, on TSA-YE plates and incubated for 48 h at 37°C. Then bacteria were harvested with a rubber policeman and suspended in 200 mL of sterile phosphate saline buffer (PBS). The bacterial suspension was centrifuged at 10,000 × g for 30 min to remove intact cells. The supernatant was passed through a 0.22 μm filter (Millipore Corp). Then, a sterility test was performed by culturing an aliquot of the filtered supernatant onto a TSA-YE plate followed by incubation for 96 h at 37°C. The sterile supernatant was centrifuged at 100,000 × g for 2 h at 4°C in an ultracentrifuge. The pellet obtained by ultracentrifugation was washed twice with 25 mL of sterile PBS and the membrane blebs were suspended in 1 mL of sterile PBS. The membrane blebs samples were divided into 0.5 mL aliquots and stored at −80°C until used.

### Protein and Lipid Quantification

The protein concentration of membrane blebs was determined using PIERCE-BCA reagents (Thermo Fisher Scientific Inc.) following manufacturer’s recommendations. The lipids of membrane blebs were measured using the lipophilic dye PKH26 (Sigma-Aldrich). Briefly, 25 μg of protein of membrane blebs were stained with 50 μL of PKH26 (diluted 1:500 with diluent C) during five min at 28°C in dark. Then, the staining was stopped adding 100 μL of bovine serum albumin (BSA) 1% in PBS (Wong-Baeza et al., 2016). The samples were washed and concentrated with microcon^®^ device (Milipore) with a cutoff of 10 kDa and finally resuspended with 200 μL of PBS. The samples were read in a fluorometer Fluoroskan Ascent FL (Thermo Fisher Scientific) at 550nm/565nm excitation/emission. The lipid quantification was reported as the fluorescent units per 25 μg of protein. The quantification of lipid and proteins was performed in triplicate.

### Observation of Membrane Blebs by Electronic Microscopy

Thirty microliter of membrane blebs (25 μg of protein) from both strains were placed onto copper grids coated with formvar and dried using filter paper. Then, 1% phosphotungstic acid was added on the samples. The grids were allowed to dry 10 h and they were observed in a transmission electron microscope (JEOL model JEM 10-10). The micrographs were taken with ATM image capture engine V. 5. 4. 2 software with different magnifications. The membrane blebs diameter was measured and the blebs were counted from ten fields with the same software. To observe membrane blebs released from the whole bacteria, cells of *B. abortus* strain 2308 and *B. abortus* strain RB51were grown on TSA plates for 36 h at 37°C, and then molten soft agar was poured to cover the growth. Once solidified the agar, small cubes (2 mm) of agar were cut. Blocks were fixed in 2.5% glutaraldehyde in PBS, rinsed with Sorensen’s PBS, dehydrated with ethanol, and prepared for transmission electron microscopy (TEM). All preparations were stained with OsO_4_, and they were observed in a transmission electron microscope (JEOL model JEM 10-10) and the micrographs were obtained as described above.

### Denaturing Gel Electrophoresis

SDS-PAGE was performed in 15% acrylamide slab gels by the method of Laemmli (1970). The gels were stained by Silver staining. The molecular masses of the proteins contained in the membrane blebs were determined by comparing their electrophoretic mobility with that of the wide range molecular mass markers (PageRuler^TM^ Prestained protein ladder, Thermo Fisher Scientific) using the computer program ImageJ v.1.49.

### Liquid Chromatography Coupled to Mass Spectrometry in Tandem (LC-MS/MS)

After the separation of the proteins contained in membrane blebs by denaturing electrophoresis, the gel was stained with Coomasie staining. Subsequently, each sample from the acrylamide gel was cut into four sections. Each section of the gel was treated with 50 mM dithiothreitol, alkylated with iodoacetamide and then “in gel” digested with trypsin. The peptides were desalted using a ZipTip^®^ (Merck KGaA, Darmstadt, German) and then concentrated in a Speed-Vac SPD 1010 Thermo-Electron (Instituto Nacional de Biotecnología-UNAM, Cuernavaca, Mexico). Each sample of membrane blebs was run in duplicate. The samples were dissolved with 50% acetonitrile containing 1% acetic acid and then placed into a Finnigan LCQ equipment. The eluate at 10 L/min was split to allow only 5% of the sample to enter the nanospray source (0.5 L/min). LC-MS/MS was performed with a PicoFrit needle/column RP C18 (New Objective, Woburn, MA, United States), using a fast gradient system from 5 to 60% of solution 100% acetonitrile with 1% acetic acid, for 45 min. The electrospray ionization source voltage was set at 1.8 kV and the capillary temperature at 130°C. Collision-Induced Dissociation (CID) was performed using 25 V of collision energy, 35–45% (arbitrary units) of normalized collision energy and the scan had the wideband activated. All spectra were obtained in the positive-ion mode. Data acquisition and the deconvolution of data were carried out using Xcalibur software on a Windows XP PC system. The MS/MS spectra from enzymatically generated peptides were analyzed by Sequest software from Finnigan (Palo Alto, CA, United States) and MASCOT search engine from Matrix Science Ltd. (Boston, MA, United States).

### *In silico* Analysis

The hits from the proteomic identification were analyzed by BlastP, using the genomic sequence of the *B. abortus* strains 2308 and RB51 (obtained from NCBI)^1^, and Uniprot^2^. Evpedia^3^ and Orthovenn^4^ analysis for enrichment of gene ontology terms were also used (Wang et al., 2015). Each protein was searched in PSORTb v. 3. 0. from ExPASy Bioinformatics Resource Portal^5^ and ProtCompB from Softberry database^6^ were used to pinpoint subcellular location of each protein. MyHits database^7^ was used to determine motif sequence on each protein.

### Protection Assay

Mice immunization was performed as previously described (Avila-Calderón et al., 2012). Briefly, female BALB/c mice of 8 weeks of age (5 per group) were vaccinated by intramuscular inoculations, at day 0 and boosted on day 30, with 5 μg of membrane blebs from virulent *B. abortus* 2308 (2308 blebs) or *B. abortus* RB51 (RB51 blebs) strains at a final volume of 50 μL adjusted with saline. One week after boosting, mice were bled from retro-orbital plexus under anesthesia (post-boost). As a positive control, a group of mice was vaccinated with 1.5 × 10^4^ CFU of vaccine strain *B. abortus* RB51. As a negative control, one group of mice was injected with saline. Mice were challenged intraperitoneally with 5 × 10^4^ CFUs of virulent strain *B. abortus* 2308 at 2 weeks post-boost vaccination. One week after challenge with the virulent *B. abortus* strain, blood was collected as before under anesthesia (post-challenge). At 2 weeks after challenge, all the mice were euthanized by CO_2_ asphyxiation followed by cervical dislocation. Spleens were collected aseptically, and CFUs/spleen of challenge strain were determined. The serum was separated from the clotted blood and stored at −20°C until use for detection of IgG subtypes.

### Indirect ELISA

Immunoglobulins IgG, IgG1, and IgG2a against antigenic proteins of 2308 membrane blebs and RB51 membrane blebs were determined in immunized mice serum post-boost and post-challenge by indirect ELISA. Sera from mice immunized with 2308 blebs was tested against purified 2308 membrane blebs and RB51 membrane blebs as antigens, while sera from mice immunized with RB51 membrane blebs was tested with 2308 membrane blebs and RB51 membrane blebs as antigens. Membrane blebs from *B. abortus* 2308 and RB51 were diluted in carbonate buffer pH 9.6 (2.5 μg/mL). The wells of 96 well polystyrene plates (Nunc-Immunoplate with maxisorp surface) were coated with 100 μL/well of membrane blebs. After incubation for 8 h at 4°C, plates were washed four times with wash buffer (Tris-buffered saline at pH 7.4, 0.5% Tween 20) and blocked with 2% bovine serum albumin (BSA) in Tris-buffered saline. After 1 h incubation at 37°C, mice sera diluted at 1:1000 in blocking buffer were added to each well (50 μL/well). The serum sample from each mouse was tested by triplicate. The plates were incubated 4 h at room temperature and then washed four times. Horseradish peroxidase-labeled anti-mouse isotype-specific conjugates (Southern Biotechnology Associates Inc., Birmingham, Alabama) were added (50 μL/well) at a dilution of 1:1000. After 1 h of incubation at 25°C, the plates were washed four times. Then, 100 μL of substrate solution (TMB Microwell peroxidase substrate; Kirkegaard and Perry Laboratories, Gaithersburg, Md) were dispensed to each well. After 20 min incubation at room temperature in the dark, the enzyme reaction was stopped by adding 100 μL of stop solution (0.185 M sulfuric acid), and the absorbance at 492 nm was recorded using microplate reader (Molecular Devices, Sunnyvale, CA, United States).

### Cytotoxicity of Membrane Blebs Through Determination of Cell Viability

The cytotoxic effect of the membrane blebs was analyzed, through an indirect cell viability analysis. Briefly, the splenocytes were cultured in a 24-well microplate with a density of 1 × 10^6^ cell/mL with supplemented media, then the splenocytes were stimulated with 1, 10, and 25 μg/mL of membrane blebs and incubated 24 h with 5% CO_2_ at 37°C. The cells were harvested, washed with sterile PBS. Phytohemagglutinin (PHA) 20 μg, was used as positive control. 1 μL of eBioscience fixable viability dye eFluor 450 (Thermo Fisher-Scientific) was added, incubated 30 min in the darkness, washed with FACS buffer, fixed with PBS containing1% paraformaldehyde (PFA), and suspended in 400 μL of FACS buffer. Samples were analyzed in LSRFortessa^TM^ cytometer (Becton-Dickinson), 30,000 events were acquired and data were analyzed with FlowJo^®^ software v. 10 (FlowJo, LLC, Ashland, Ore).

### Evaluation of Activation Surface Markers by Flow Cytometry

Three mouse groups were included in this assay, with three animals per group. The first group was immunized with 5 μg of purified 2308 membrane blebs, a second group was immunized with 5 μg of purified RB51 membrane blebs, and the third group was injected with saline by intramuscular route. Three days after boosting, all mice were euthanized by CO_2_ asphyxiation followed by cervical dislocation, and the spleens were collected aseptically. Spleens were mashed with 5 mL of sterile PBS on a cell strainer (Corning^®^) and splenocytes were collected in a 50 mL sterile tube. Then, 10 mL of lysis solution (KHCO_3_-NH_4_Cl-EDTA) was added, mixed and kept on ice for 8 min. Subsequently, 25 mL of cold sterile PBS was added and centrifuged 10 min, 1200 rpm at 4°C. The cells were placed in RPMI medium supplemented with 10% fetal calf serum (FCS), 100 IU penicillin/100 μg/mL streptomycin, and 2.5 μg/mL fungizone. Cells were counted by trypan blue exclusion assay. Two mL of a suspension of 1 × 10^6^ spleen cells/mL was added to each well in a 24-well plate (in duplicate). Spleen cells coming from mice immunized with 2308 membrane blebs, RB51 membrane blebs or mice injected with saline were stimulated with either 5 μg of 2308 membrane blebs, 5 μg RB51 membrane blebs, or 5 μg of *Salmonella* lysate. Plates were incubated 24 h, at 37°C under 5% CO_2_.

After stimulation, spleen cells were collected and washed with FACS buffer (PBS, 1% FCS, sodium azide 0.01%) and incubated with diluted mAbs in FACS buffers: anti-mouse CD19-APC, anti-mouse CD3-FITC, anti-mouse CD4-PerCP, anti-mouse CD69-PE, anti-mouse CD11c-APC, anti-mouse CD86-PE, and anti-mouse MHC-II (I-A/I-E)-FITC for 1 h at 4°C protected from the light. Then, the cells were washed with FACS buffer, fixed with PBS containing 1% paraformaldehyde (PFA), and suspended in 400 μL of FACS buffer. Samples were analyzed in LSRFortessa^TM^ cytometer (Becton-Dickinson), 500,000 events were acquired and data were analyzed with FlowJo^®^ software v. 10 (FlowJo, LLC, Ashland, Ore).

### Identification of Antigenic Proteins in the Membrane Blebs

30μg of blebs obtained from *B. abortus* 2308 and RB51 were loaded in each well of an 15% SDS-PAGE. A wide range molecular mass markers was included (PageRuler^TM^ Prestained protein ladder, Thermo-Fisher Scientific). After running the gel was transferred to a PVDF membrane (Inmobilon-P Millipore^®^) in a semidry chamber for 30 min at 20 V. The PVDF membrane was washed with TBS-Tween 20 (0.05%) during 5 min. The membrane was blocked with 5% low fat dry milk in TBS-Tween 20 during 2 h at room temperature. Then, the membrane washed three times with 10 mL of TBS-Tween 20. After that, the serum of immunized mice diluted in TBS-Tween 20 (1:5000) was added and incubated 2 h at room temperature. The membrane was washed three times, and incubated with a secondary antibody Anti-Mouse IgG (whole molecule) coupled to peroxidase (Sigma-Aldrich) diluted 1:5000 with TBS-Tween 20 during 1 h at room temperature. Finally, the membrane was washed three times and revealed with Inmobilon Western kit (Millipore^®^), the images were obtained with a Gel Doc system (Bio-Rad) and the Image Lab^TM^ software (Bio-Rad).

### Ethics Statement

All the mice experiments were done in AAALAC approved facility and the mice experimental protocols were approved by the Institutional Animal Care and Use Committee (IACUC) (protocol # CVM-10-048) at Virginia Tech, which follows protocols approved by the American Veterinary Medical Association (AVMA). For retro-orbital bleeding, mice were anaesthetized under isofluorane using Vet Equip Mobile Laboratory Animal Anesthesia System. Mice were euthanized using an overdose of carbon dioxide followed by cervical dislocation.

### Statistics

Two-way ANOVA analysis with Bonferroni post-tests was performed for analysis of the mean fluorescence intensity of the surface markers expression and IgG subtypes by ELISA, and One-way ANOVA analysis with Tukey post-test was used to compare results from protection experiments and analysis of the surface marker activation in the splenocytes. An unpaired t test with 95% confidence interval was performed to analyze the results obtained for protein and lipid quantification and membrane blebs counting. GraphPad Prism v.5.01 software was used for the statistical analyzes.

## Results

### Obtaining Membrane Blebs From *B. abortus* Strains

**FIGURE 1:**
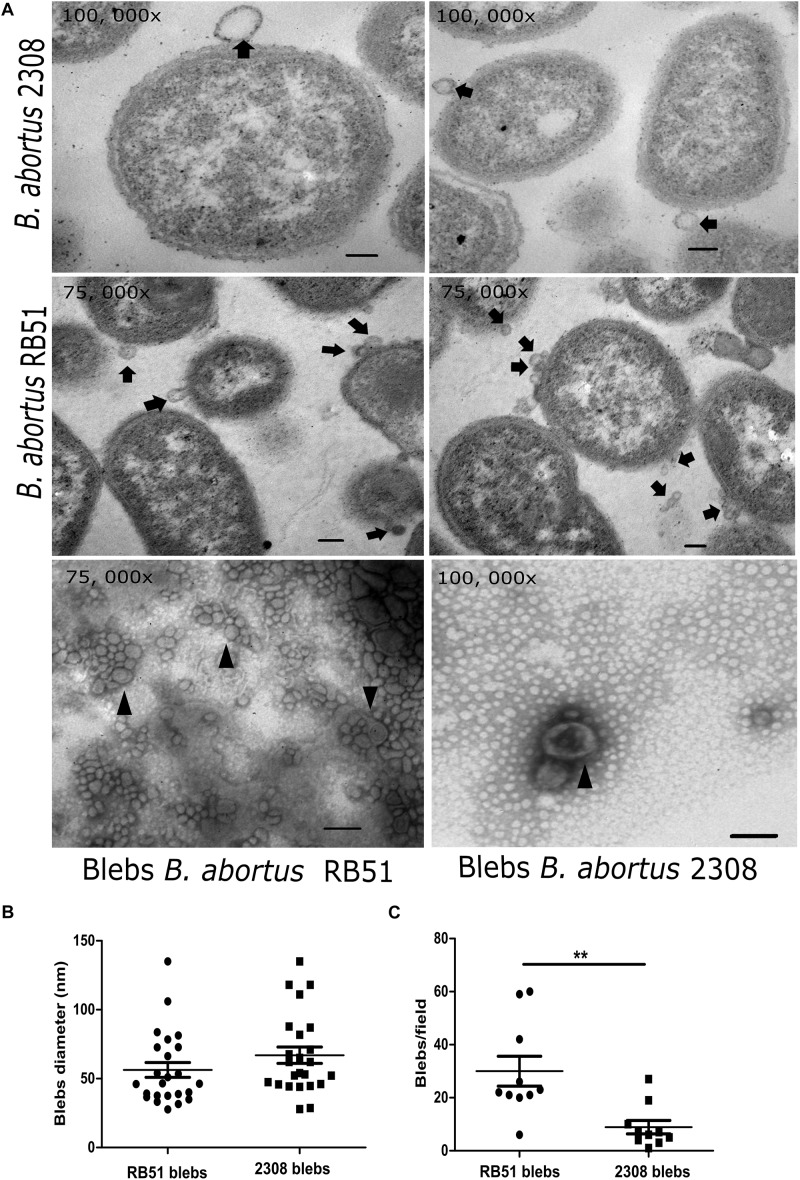
Electron microscopy of *B. abortus* membrane blebs. **(A)** Agar embedded whole bacteria was processed for the thin section and negatively stained with OsO_4_. In the electron micrographs, blebs are pinched off from the bacterial surface (black arrows). Membrane blebs stained with phosphotungstic acid showed vesicles with a double membrane (arrowheads). Membrane blebs showed ranging in from size from 29.7 to 135 nm in the virulent strain, while in the vaccine strain it ranged from 27.7 to 135 nm. **(B)** Membrane blebs counted from ten fields from both strains (*P* = 0.0031; ^∗∗^*P* < 0.05). **(C)** Diameters in nm of the membrane blebs from both *B. abortus* strains measured with AMT image capture engine V. 5. 4. 2 software. Bar = 100 nm.

In the micrographs, the membrane blebs released from the whole cell of *B. abortus* 2308 and RB51 were observed (Figure 1A). Membrane blebs from *B. abortus* 2308 showed sizes from 27.9 to 135 nm with an average of 66. 9 nm, while the membrane blebs from the vaccine strain showed blebs with sizes from 27.7 to 135 nm with an average of 56.27 nm (Figure 1B). In both strains, membrane blebs showed a spherical shape and a bilayer-lipidic membrane. In several electron microscope images, we noticed that *B. abortus* RB51 strain released more membrane blebs than the *B. abortus* 2308, in a ratio of 30:8.9 membrane blebs per field (unpaired t test with 95% confidence) (Figure 1C). Regarding the protein and lipid quantitation, an average of 1.421 ± 3. 6 mg/mL of protein for 2308 membrane blebs and 1.377 ± 9.6 mg/mL for RB51 membrane blebs was obtained, but the differences were not statistically significant (unpaired t test with 95% confidence). In the case of the lipid quantification, it an average of 61.18 ± 7.8 fluoresce units for 2308 blebs and 102.6 ± 7.9 fluoresce units for RB51 membrane blebs (*P* < 0.05) (unpaired *t*-test with 95% confidence) was obtained. These quantification, indicated that *B. abortus* RB51 produced more blebs than the *B. abortus* 2308.

### Proteomic Analysis

**FIGURE 2:**
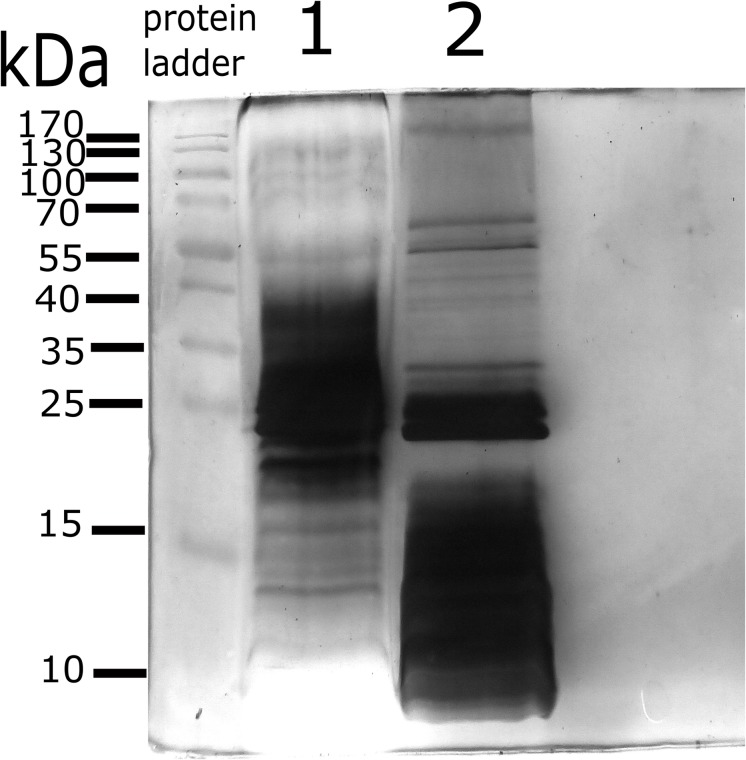
Protein profile of membrane blebs from *B. abortus* 2308 and the vaccine strain RB51. Blebs were obtained by differential centrifugation and loaded onto 15% Acrylamide gel for electrophoresis. Gel stained by Silver staining. **Lane 1**; proteins profile of membrane blebs from *B. abortus* 2308. **Lane 2**; proteins profile of membrane blebs from *B. abortus* RB51. 45 μg of the protein of membrane blebs were loaded onto each well. Protein Ladder; PageRuler^TM^ Prestained protein ladder, Thermo-Fisher Scientific.

The electrophoretic protein profile showed proteins from 10 to 92 kDa for 2308 membrane blebs and 10 to 139 kDa for RB51 membrane blebs (Figure 2). After electrophoretic separation, proteomic analysis was performed in order to identify the protein composition of membrane blebs of *B. abortus*. The resulting peptide sequences were used to query databases that led to the identification of 271 hits for the *B. abortus* 2308 strain and 214 hits for *B. abortus* RB51 strain. A query result was only considered as significant if the overall score was higher than 20 and more than two tryptic peptides as well as their fragment ions matched to the protein and the calculated molecular weight corresponded to the molecular weight in the original gel section (Avila-Calderón et al., 2012).

**FIGURE 3:**
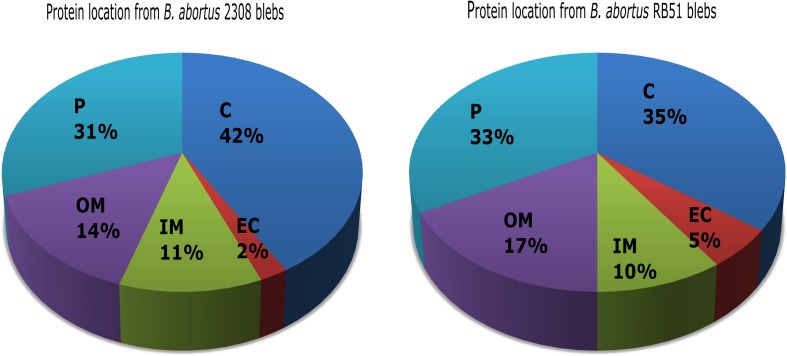
Protein *in silico* analysis from *B. abortus* membrane blebs. Subcellular locations of blebs proteins using PSORT3b and Softberry database. Periplasmic (P), cytoplasmic (C), outer membrane (OM), inner membrane (IM), and extracellular (EC) locations are indicated as mentioned.

These aforementioned hits were analyzed with BlastP using the genome of *B. abortus* RB51-AHVLA strain from the NCBI database. Hits from the 2308 membrane blebs strain were analyzed with Uniprot database against *B. abortus* 2308 genome. The hits that were unambiguously identified in the genome were 228, meanwhile 171 were identified as unique proteins for membrane blebs from *B. abortus* 2308 and *B. abortus* RB51 respectively. The rest of the sequences were discarded from further analysis. The identified proteins from both *B. abortus* strains were annotated according to aminoacid length, molecular weight, isoelectric point, locus tag, subcellular location, COG functional classification and protein domains (Supplementary Tables S1, S2). *In silico* analysis revealed that proteins identified in 2308 membrane blebs were 42% cytoplasmic, 31% periplasmic, 14% outer membrane, and 11% inner membrane. In the case of RB51 membrane blebs, the proteins were 35% cytoplasmic, 33% periplasmic, 17% outer membrane, and 10% inner membrane (Figure 3).

Analysis of orthologous proteins was performed to identify the overlapping orthologous clusters in the membrane blebs from both strains. The analysis showed that proteins from 2308 membrane blebs were classified into 115 clusters, while proteins from RB51 membrane blebs were classified into 113 clusters. From these clusters, 112 were shared in blebs from both tested strains, whereas 3 clusters were unique for membrane blebs from *B. abortus* 2308 and 1 was unique for RB51 membrane blebs. On the other hand, 108 proteins from 2308 membrane blebs were not classified into the orthologous clusters (singletons) in the same way 57 proteins were found in RB51 membrane blebs.

From the 112 orthologous clusters shared between both strains, the proteins were grouped based on their biological process ontology into 33 groups and 13 based on their molecular function ontology (Supplementary Figures S1A,B). The most important groups based on biological process ontology were translation (GO: 0006412), biological process (GO: 0008150), metabolic process (GO: 0008152), and cellular process (GO: 0009987). The most important groups based on molecular function process were structural molecule activity (GO: 0005198), nucleic acid binding (GO: 0003676), binding (GO: 0005488) nucleotide and nucleoside binding (GO: 0000166, GO:0001882) and ion binding (GO: 0043167) (Supplementary Figure S1A). The prevalence of some proteins in membrane blebs from both strains was not unexpected, for example, SodC, Omp25, Omp31-1, Omp19, and Omp16, since these were also found previously in membrane blebs purified from *B. melitensis* 16M and *B. suis* 1330 (Boigegrain et al., 2004; Avila-Calderón et al., 2012).

### Immune Protection Assays

**FIGURE 4:**
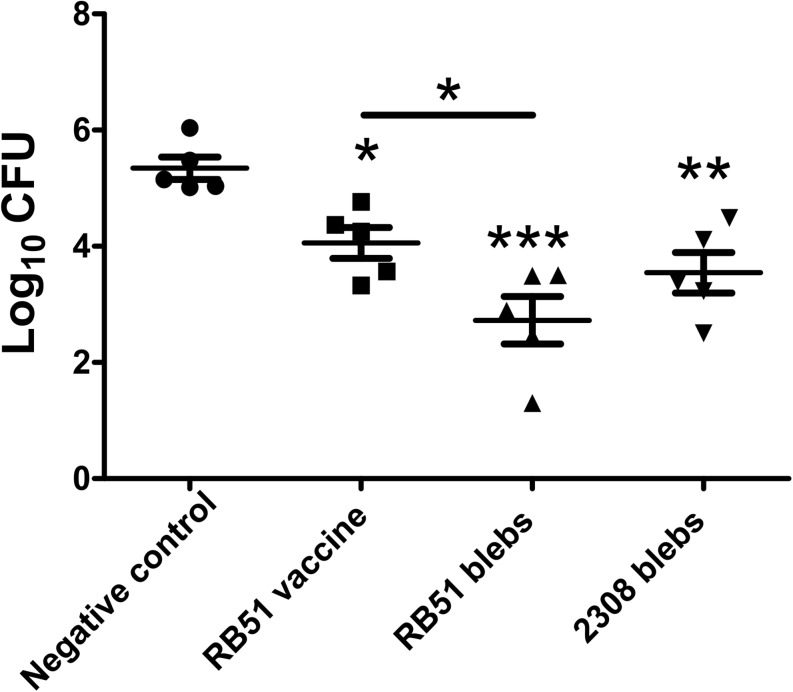
Protection assay in mice immunized with membrane blebs from *B. abortus* strains. Impact of vaccination on bacterial burdens after intraperitoneal challenge with virulent *B. abortus* 2308. Mice were immunized intramuscularly with 5 μg of proteins from membrane blebs and boosting 1 month later with the same amount of blebs. Control group were vaccinated with live vaccine *B. abortus* RB51 and a negative control with saline. ^∗^*P* < 0.05, *^∗∗^P* < 0.01, *^∗∗∗^P* < 0.001.

Mice immunized with membrane blebs from *B. abortus* 2308 and *B. abortus* RB51 were challenged with *B. abortus* 2308. Protection was defined as a significant reduction in bacterial burden in the spleens of mice immunized with membrane blebs, found 2 weeks post-challenge. The group of mice immunized with the vaccine strain *B. abortus* RB51 induced 1.288 log units of protection compared to saline control (*P* < 0.05) (one-way ANOVA with Tukey’s Multiple Comparison Test, confidence interval of 95%). In the case of mice vaccinated with membrane blebs from *B. abortus* 2308 induced 1.8 log units (*P* < 0.01), while membrane blebs from *B. abortus* RB51 induced 2.618 log units of protection compared to saline control (*P* < 0.001) (Figure 4).

### Determination of IgG Antibodies in Mice Immunized With *B. abortus* Blebs

**FIGURE 5:**
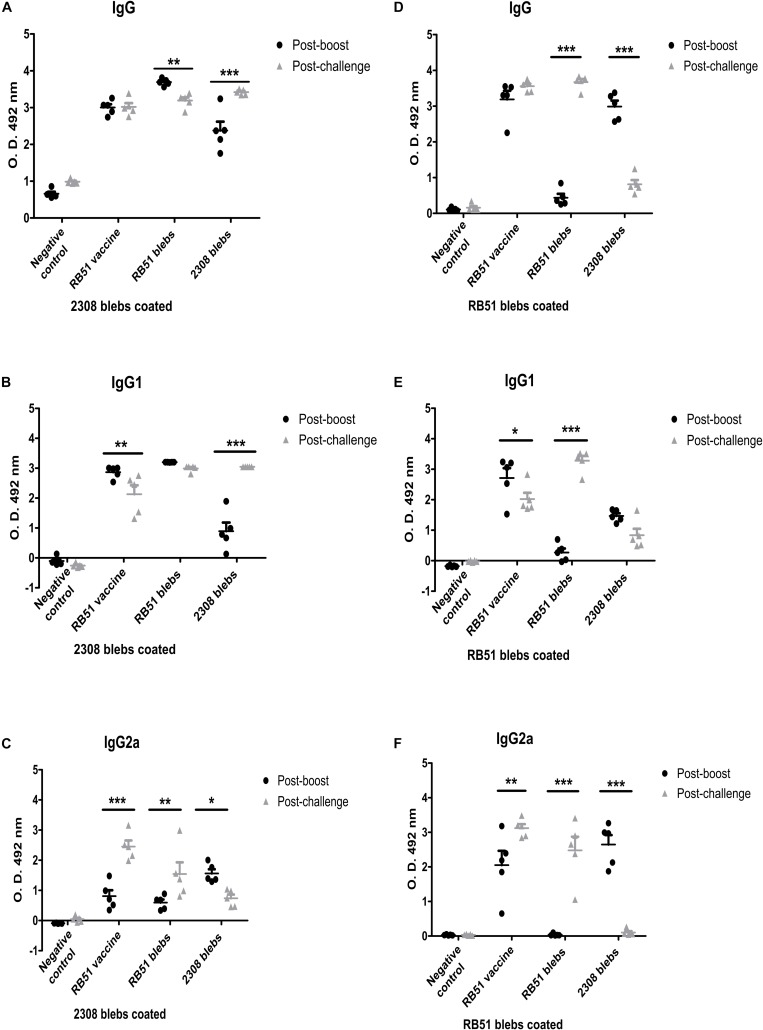
Analysis of IgG antibody responses of BALB/c mice to blebs from *B. abortus*. Membrane blebs were obtained from *B. abortus* 2308 and RB51, and mice were immunized as described above (Figure 4). Sera from each mouse were collected and were assayed individually by ELISA. Antibody levels are expressed as optical density (OD) at 492 nm. **(A–C)** Indirect ELISA for quantification of IgG, IgG1, and IgG2a from immunized mice with coated plates with 2.5 μg/mL of proteins of membrane blebs from *B. abortus* 2308. **(D–F)** Indirect ELISA to quantification of IgG, IgG1, and IgG2a from immunized mice using coated plates with 2.5 μg/mL of proteins of membrane blebs from *B. abortus* RB51. *^∗^P* < 0.05, *^∗∗^P* < 0.01, *^∗∗∗^P* < 0.001.

In this work the IgG subtypes were evaluated, because it is known that IFNγ (Th1 cytokine) and IL-4 (Th2 cytokine) induce isotype switching to IgG2a and IgG1, respectively. Therefore, different IgG isotype levels could support how the immune response is directed. This methodology has been used in other reports, being a valid assay to determine indirectly the Th1 or Th2 response (Snapper and Paul, 1987; Finkelman et al., 1990; Rostamian et al., 2017). To evaluate the induction of specific and cross-reacting antibodies against membrane blebs, sera from mice immunized with 2308 blebs were tested against 2308 membrane blebs and RB51 blebs as antigens. Similarly, sera from mice immunized with RB51 membrane blebs were tested against 2308 blebs and RB51 blebs as an antigens. The quantification of the antibodies was performed in serum collected 1 week after boosting and 1 week after the challenge with the virulent strain *B. abortus* 2308. After the challenge with the virulent *B. abortus*, mice immunized with 2308 blebs increased the total IgG and IgG1 levels to a greater extent than mice vaccinated with the live vaccine, when wells were coated with blebs 2308 (*P* < 0.001) (Two-way ANOVA with Bonferroni post-tests, confidence interval of 95%). Also, high levels of IgG2a were observed in the mice group immunized with 2308 membrane blebs after the boost, when wells were coated with blebs 2308 or blebs RB51 (*P* < 0.05) (Figures 5A–C). Mice immunized with RB51 membrane blebs did not induce significant levels of total IgG, IgG1, and IgG2a against RB51-antigens after the boost. The highest levels of antibodies against RB51 antigens from mice immunized with RB51 membrane blebs was observed after the challenge with the virulent *B. abortus* strain (*P* < 0.001) (Figures 5D–F). On the other hand, the immunization with membrane blebs from *B. abortus* 2308 induced the highest levels of IgG2a against RB51 antigens mainly after the boost (*P* < 0.001) (Figures 5D,F). The mice vaccinated with the live vaccine strain RB51, the antibodies induced were probably against the common antigens present in both membrane blebs. In this group of mice, the levels of antibodies against 2308 membrane blebs antigens were higher after the challenge, whereas the levels of antibodies against RB51 membrane blebs antigens were not different at two times measured (Figures 5A–F).

### Cytotoxicity of Membrane Blebs Through Determination of Cell Viability

The cytotoxic effect of the blebs was analyzed in splenocytes, through an indirect cell viability analysis. Results showed that different concentrations of membrane blebs from both strains had no effect on the cell viability of splenocytes at 24 h (Supplementary Figure S2). Through this experiment it could be possible to demonstrate that *Brucella* membrane blebs are not cytotoxic for splenocytes.

### Evaluation of Activation Surface Markers Induced by *B. abortus* Membrane Blebs

**FIGURE 6:**
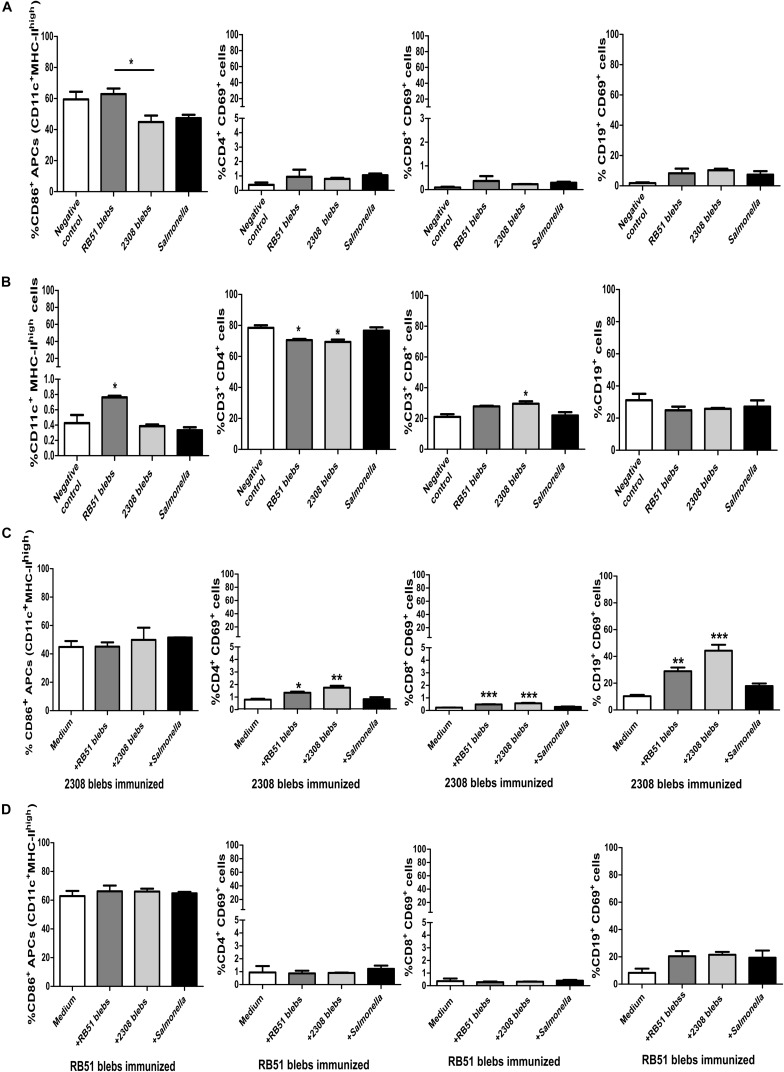
Analysis of expression of surface markers on lymphocytes and APCs from mice immunized with *B. abortus* membrane blebs. **(A)** Activation was measured by flow cytometry using mAbs against surface molecules (CD86) on APCs. The percentage of APCs and T CD3^+^ cells was measured to evaluate possible changes in the percentages of these populations. T helper lymphocytes (CD3^+^CD4^+^) were gated to analyze individually the expression of surface molecule CD69. Cytotoxic T cells were gated as the exclusion of the CD3^+^CD4^–^. **(B)** The total of APCs and T and B cells also was measured. **(C)** Spleen cells from 2308 membrane blebs immunized mice were cultured with 5 μg of membrane blebs from either *B. abortus* strain membrane blebs during 24 h. **(D)** Spleen cells from RB51 blebs immunized mice were cultured with 5 μg of blebs from either *B. abortus* strain blebs during 24 h *^∗^P* < 0.05, *^∗∗^P* < 0.01, *^∗∗∗^P* < 0.001.

During the acute phase of brucellosis, the number of CD4^+^ and CD8^+^ T cells at the spleen decrease and, the main antibodies producing cells, the B-lymphocytes CD19^+^, are located also in this organ (Grilló et al., 2012). Therefore, it was expected to have a decrease in cells (at least in the lymphocytic population) in the negative control mice, which were not vaccinated with *Brucella* blebs, 2 weeks post-challenge with *B. abortus* 2308. Interestingly, mice immunized with RB51 membrane blebs increased the percentage of total APCs, whereas immunization with 2308 blebs increased the percentage of total T CD3^+^CD8^+^ cells compared with the negative control group (Figures 6A,B) (*P* < 0.05) (one-way ANOVA with Tukey’s Multiple Comparison Test, confidence interval of 95%).

Stimulation of splenocytes from immunized mice with 2308 membrane blebs increased the percentage of T helper, cytotoxic and B cells expressing the activation surface marker CD69 (Figure 6C) (*P* < 0.05) (one-way ANOVA with Tukey’s Multiple Comparison Test, confidence interval of 95%). There was a slight increase in the APCs and B cells after stimulation with RB51 membrane blebs, but this difference was not statistically significant (Figure 6D) (one-way ANOVA with Tukey’s Multiple Comparison Test, confidence interval of 95%).

Immunization with membrane blebs from both strains did not affect the expression of CD86 molecules since Mean Fluorescence Intensity (MFI) values did not change significantly compared with mice injected with saline. Also, the MHC-II MFI values decreased drastically in mice immunized with RB51 membrane blebs while CD11c expression decreased in mice immunized with membrane blebs from both *Brucella* strains, so these results were merely based on activated cells but not by up- or down-regulation of molecules analyzed (Supplementary Figure S3A) (Two-way ANOVA with Bonferroni post-test, confidence interval of 95%). The expression (MFI) of the CD4 or CD69 molecules in the T helper cells or CD3 or CD69 in the cytotoxic T cells were not affected, whereas the CD19 expression decreased in mice immunized with membrane blebs (Supplementary Figures S3B–D) (Two-way ANOVA with Bonferroni post-test, confidence interval of 95%).

### Identification of Antigenic Proteins in the Blebs

The total antibodies of serum from immunized mice with membrane blebs of *B. abortus* 2308, recognized a band of approximately 23 kDa in the western blot. While the serum from immunized mice with blebs of *B. abortus* RB51 recognized proteins from 10 to 70 kDa (Supplementary Figure S4). Also, in the western blot it is possible to observe that antibodies bound to LPS, just in the sample of the membrane blebs from *B. abortus* 2308 that is a smooth strain, but no in the rough strain RB51.

## Discussion

The release of membrane blebs from the surface of Gram-negative bacteria is still an intriguing bacterial process. These membrane blebs carry cellular components from the progenitor cell naturally; this makes them useful to induce an immunological response *in vitro* and *in vivo* without being infectious like the complete cell. Based on these features membrane blebs from different pathogens have been tested as an acellular vaccine in animal models and more recently they have been proposing as a target for drug delivery (Tan et al., 2018). Herein for the first time, we describe the complete proteomic profile of membrane blebs from *B. abortus* virulent strain 2308 and the vaccine *B. abortus* RB51 and tested as an acellular vaccine *in vivo*. *B. abortus* is the etiologic agent of the bovine brucellosis, a zoonotic disease that causes public health and economic losses issues. Previously, Pollak et al. (2012) purified membrane blebs from *B. abortus* strain 2308 reporting membrane blebs ranging in sizes from 30 to 130 nm with an average of 85 nm. Later, Kaur et al. (2016) isolated membrane blebs from *B. abortus* strain 99 observing membrane blebs from 20 to 300 nm. In this work, the sizes of membrane blebs observed for 2308 membrane blebs were from 27.9 to 135 nm and from 27.7 to 135 nm for the strain RB51.

In the last decades, proteomics became a crucial tool to reveal the protein composition, being the 1D-SDS-PAGE coupled to the liquid chromatography and mass spectrometry the most common methodology to know the content of membrane blebs (Lee et al., 2016). Through proteomic analysis of membrane blebs isolated from *B. abortus* 2308 and RB51 were identified proteins such as SodC, Omp16, Omp19, Omp31-1, and Omp25, which have been previously reported as virulence factors or immunogenic proteins in *Brucella*. Also, these proteins were part of the 112 orthologous proteins shared in membrane blebs from 2308 and RB51 strains. These proteins showed similar distribution in the subcellular location in both strains. Based on the analysis of the constituent proteins in the membrane blebs we could estimate that at least 49% of the proteins were common in both strains tested in this work. It was expected to find some similarities between both strains since the RB51 vaccine is a rough mutant derivated from the 2308 strain; RB51 strain has an *IS711* insertional sequence in the *wboA* gene (Moriyón et al., 2004). The *wboA* gene encodes for a glycosiltransferase, essential for the biosintesis of the O-side chain (McQuiston et al., 1999). Previous reports have demonstrated that O-side chain lacking in the LPS alters protein cargo of membrane blebs. For example, deletion of the galactosyltransferase gene *wbbO* of *Klebsiella pneumoniae* leads to O-side chain lacking phenotype, this modification altered the protein composition of membrane blebs compared with membrane blebs from the wild-type, without affecting the release of blebs or the cell growth (Cahill et al., 2015). The lack of O-side chain likely affects protein packing of membrane blebs from the vaccine strain in number, type of proteins and reflects slightly different metabolic pathways enrichment likewise in *K. pneumoniae wbbO* mutant. In the case of the wildtype *K. pneumoniae*, their membrane blebs were enriched with proteins involved in the cell wall, membrane, and envelope biogenesis, whereas vesicles purified from *K. pneumoniae wbbO* mutant contained proteins involved in the post-translational modification, protein turnover, and chaperones. In the case of *B. abortus* 2308, its membrane blebs were enriched with proteins involved in the environmental processing information, carbohydrate metabolism, amino acid metabolism, and genetic processing information. Whereas, membrane blebs from *B. abortus* RB51 were enriched with proteins involved in lipid metabolism, cellular process, and genetic information processing (Supplementary Figure S1C). It has been proposed that proteins bound to a negatively charged O-side chain leading to protein packing into the membrane blebs, however, in some enterobacteria, negative charge come from the core, rather than O-side chain (Frirdich and Whitfield, 2005; Bonnington and Kuehn, 2014). In *B. melitensis* the core oligosaccharide branch, not linked to the O-antigen, has a positive charge and balances the negative internal charges (Fontana et al., 2016). We suggest that O-side chain lacking, inbalance in their charges at the surface and this affects protein packing into the RB51 membrane blebs.

In the last decades an effort has been made to improve vaccines against brucellosis, so the research focus has been to test bacterial extracts, pure recombinant proteins, mutant strains, or DNA vaccines in animal models. Omp16, Omp19, Omp25, Omp31, and SodC have been described as immunogenic and protective antigens against brucellosis (Avila-Calderón et al., 2013). However, these proteins and others tested as vaccines have not elicited the level of the cellular response required to eliminate the intracellular bacteria, not like the one induced by a live *Brucella* vaccine. Previously, Omp19, Omp25, Omp31, and SodC were identified in *B. melitensis* membrane blebs by proteomics, also these membrane blebs were able to protect mice against the challenge with *B. melitensis* 16M (Avila-Calderón et al., 2012). The report of *B. melitensis* membrane blebs was the first evidence about the use of membrane blebs as a vaccine against brucellosis, which elicited a cellular protective response in mice, similar to that induced by the *B. melitensis* Rev1 live vaccine. This finding was particularly important because currently the *Brucella* vaccines are made with live cells, so the use of membrane blebs would avoid the use of *Brucella* whole cell and would be much safer. In this work, membrane blebs obtained from *B. abortus* 2308 and RB51 contain Omp16, Omp19, Omp25, Omp31, SodC among others identified by proteomics, as we mentioned before, these proteins were previously described as antigenic and protective proteins in *Brucella*. The results of the western blot showed that, the proteins recognized by the antibodies in the serum of immunized mice corresponded to the group 3 of outer membrane proteins of *Brucella*, based on the molecular weight, probably Omp25/Omp31.

Membrane blebs from the *B. abortus* 2308 and RB51 were used to evaluate protection and immunological antibody response in mice. In this work we evaluated the IgG subtypes, because it is known that IFNγ (Th1 cytokine) and IL-4 (Th2 cytokine) induced isotype switching to IgG2a and IgG1, respectively. Therefore, different IgG isotype levels could support how the immune response is drive. This methodology has been used in other reports, being a valid assay to determine indirectly the Th1 or Th2 response (Snapper and Paul, 1987; Finkelman et al., 1990; Rostamian et al., 2017). Membrane blebs from both strains demonstrated the ability to induce protection and reduce bacterial burden at a similar level compared to the live vaccine *B. abortus* RB51 (*P* < 0.05). Moreover, IgG antibodies were measured post-boosting and post-challenge (Figure 5). It was expected to find cross-reaction of IgG antibodies tested positive against 2308 membrane blebs and RB51 membrane blebs since the rough vaccine RB51 strain is a derivate from the virulent strain 2308. As previously mentioned, several proteins were common in membrane blebs from both strains. Mice immunized with the live vaccine and with membrane blebs from either of the strains, induced total IgG, IgG1 and IgG2a antibodies against 2308 membrane blebs antigens at post-boosting, whereas the levels of antibodies against RB51 membrane blebs were more elevated after the challenge with the virulent *B. abortus* 2308. Mice immunized with 2308 membrane blebs induced IgG and IgG1 anti-RB51 antigens post-boosting and post-challenge, but only induced specific IgG2a antibodies after boosting. On the other hand, humoral immune response elicited by immunization with membrane blebs from the RB51 strain was almost exclusively against antigens from the same blebs and elicited high levels of IgG1 after the challenge. Clearly, 2308 membrane blebs induced better humoral response since IgG1 and the IgG2a response was elicited after the boosting and remained high post-challenge. Although IgG1 response elicited by RB51 membrane blebs immunization was higher post-challenge, it was likely due to the antibodies level induced by the challenge itself instead of vaccination, since specific IgG1 anti-RB51 membrane blebs were not observed post-boosting. Moreover, the blood sample was taken 1 week after the challenge when the infection is in the acute phase. The levels of IgG antibodies induced by 2308 membrane blebs and RB51 membrane blebs (OD values higher than 3) were higher than those reported by Kaur et al. (2016) elicited by membrane blebs from the *B. abortus* strain 99 (OD values lower than 2). However it is important to mention that methodology followed by Kaur et al., and the one followed in this work showed substantial differences. Previously, it was observed similar and slightly high (OD values higher than 5 for IgG2a) values of antibodies in immunized mice with membrane blebs from *B. melitensis* (Avila-Calderón et al., 2012). Antibodies promote host defense as direct or indirect effector molecules for the immune response by complement activation or interacting with FcR receptors on the phagocytes and modulating the release of cytokines. Although it was observed a high humoral immune response in mice vaccinated with *Brucella* membrane blebs, the humoral response is not considered the most critical component of the immune response to control intracellular bacteria (Casadevall and Pirofski, 2006).

In this work, analysis of the immune response induced by membrane blebs in lymphocytes and APCs from mice spleens was performed. During the acute phase of brucellosis, the number of CD4^+^ and CD8^+^ T cells at the spleen decreased and, the main antibody producing cells, the B-lymphocytes CD19^+^, also reside in the spleen (Grilló et al., 2012). Therefore, it was expected to have a decrease in cell number (at least in the lymphocytic population) in the negative control mice, which had not been vaccinated with *Brucella* membrane blebs, 2 weeks post-challenge with *B. abortus* 2308, due to the effect of challenge. We observed that the splenocytes from mice immunized with 2308 membrane blebs were able to increase the percentage of total activated cells (lymphocytes) after the treatment with 2308 or RB51 membrane blebs, while the splenocytes from mice immunized with membrane blebs of *B. abortus* RB51 did not. Similar results were observed by Stevens et al. (1994). In this experiment, mice were inoculated with *B. abortus* 2308, RB51 and S19, and splenocytes were obtained from mice at 6, 10, and 20 weeks post-infection. Splenocytes were subsequently treated with protein fractions of a lysate of *B. abortus* (22 fractions from 18 to 106 kDa) to evaluate lymphoproliferation at day 5. Authors observed lymphoproliferation in cells (from mice at 10, and 20 weeks post-infection) treated with fraction of 18 kDa, except in cells coming from mice inoculated with *B. abortus* RB51 (at week 20). Based on knowing that membrane blebs are constituted of cellular components but not virulent like the whole cell, and taking into account the protection results observed in this work, *Brucella* membrane blebs position themselves as new model to research development of acellular vaccines against brucellosis.

Interestingly, stimulation of the splenocytes from immunized mice with 2308 membrane blebs or RB51 membrane blebs decreased the expression of MHC-II, being more evident in mice immunized with RB51 blebs (Supplementary Figure S2A). Pollak et al. (2012) also observed the down-regulation of MHC-II induced by IFN-γ in vitamin D3-differentiated THP-1 cells stimulated with membrane blebs from *B. abortus* 2308. Omp19 was identified in the 2308 membrane blebs and RB51 membrane blebs, this protein induces the secretion of IL-6 decreasing the transcription of the master regulator of MHC-II, thus downregulating MHC-II expression (Velásquez et al., 2017). It is noteworthy that T cytotoxic cells percentage increased on mice immunized with membrane blebs from both strains. Kaur et al. (2016) observed lymphoproliferation in mice immunized with membrane blebs from *B. abortus* 99S higher than the negative control group but lesser than the cells stimulated with concanavalin A (Kaur et al., 2016). On the other hand, in this work, the immunization and stimulation of splenocytes with 2308 membrane blebs increased significantly the percentage of activated B cells. Previously, membrane blebs purified from *B. melitensis* VTRM1, a rough strain, induced high expression levels of Th1 cytokines genes such as IL-12, TNFα, and IFNγ in bone-marrow derived dendritic cells (BMDC) (Avila-Calderón et al., 2012).

In this work, immunization with membrane blebs from both *B. abortus* strains induced protective immune response against virulent *B. abortus* challenge. Likely, the protective immune response elicited by *B. abortus* membrane blebs immunization rely on the action of professional phagocytes such as macrophages or neutrohils rather than APCs. Clearly, immunization with *B. abortus* 2308 membrane blebs induced cellular and humoral immune response, since stimulation of the splenocytes induced increased levels of T and B cells expressing CD69 and strong IgG2a immune response after the boost. However, we propose the use of membrane blebs from the live vaccine *B. abortus* RB51: (i) first, immunization with RB51 membrane blebs induced 1.33 log units of protection more than the whole live vaccine strain which is approved for their use in animals; (ii) the membrane blebs, from a rough strains like the RB51 strains do not induce antibodies against *Brucella*-LPS, therefore, do no interfere with the serological diagnostic; (iii) although 2308 membrane blebs induced strong IgG response, this could be detrimental in chronic brucellosis phase. In fact, infection with smooth *Brucella* strains induced high titers of antibodies against LPS, whereas immunization of cattle with RB51 vaccine induced IgG antibodies but not against *B. abortus* 2308-LPS (Stevens and Olsen, 1996; Letesson et al., 1997).

In conclusion: Membrane blebs from *B. abortus* 2308 and RB51 induced protection similarly than the live vaccine *B. abortus* RB51 in mice, membrane blebs offer the advantage of being non-virulent as live *Brucella* vaccines and contain antigenic and cellular components able to induce protective response. This work contributes to the current knowledge of the *Brucella* membrane blebs, showing encouraging results concerning protection provided by blebs, specially membrane blebs derived from the rough strain RB51, against *Brucella* challenge infection.

## Data Availability Statement

All datasets generated for this study are included in the article/Supplementary Material.

## Ethics Statement

All the mice experiments were done in AAALAC approved facility and the mice experimental protocols were approved by the Institutional Animal Care and Use Committee (IACUC) (protocol # CVM-10-048) at Virginia Tech, which follows protocols approved by the American Veterinary Medical Association (AVMA). For retro-orbital bleeding, mice were anaesthetized under isofluorane using Vet Equip Mobile Laboratory Animal Anesthesia System. Mice were euthanized using an overdose of carbon dioxide followed by cervical dislocation.

## Author Contributions

MA-V, EA-C, AC-R, FS-G, and LF-R conceived and designed the experiments. MA-V, EA-C, AC-R, MR-P, JC-A, NS, HQ, SW, and ZG-L performed the experiments. MA-V, EA-C, ER, and MA-A analyzed the data. EA-C, MA-A, MR-P, LF-R, and AC-R wrote the manuscript. LF-R and MA-A gave practical suggestions to perform the experiments.

## Conflict of Interest

The authors declare that the research was conducted in the absence of any commercial or financial relationships that could be construed as a potential conflict of interest.

## Footnotes

1 https://www.ncbi.nlm.nih.gov/protein/?term=brucella+abortus+rb51

2 https://www.uniprot.org/uniprot/?query=brucella+abortus+2308&sort=score

3 http://student4.postech.ac.kr/evpedia2_xe/xe/

4 http://www.bioinfogenome.net/OrthoVenn

5 http://www.psort.org/psortb/index.html

6 http://linux1.softberry.com/berry.phtml?topic=pcompb&group=programs&subgroup=proloc

7 https://myhits.isb-sib.ch/

## Supplementary Material

FIGURE S1 Enrichment of function for orthologous proteins identified in *B. abortus* membrane blebs. Distributions of *B. abortus* specific 112 orthologous protein sets in Molecular function **(A)** and biological process **(B)** GO slim terms. **(C)** Enrichment metabolic pathways of *B. abortus* 2308 and RB51 membrane blebs proteins. The proteins identified were sorted according to the indicated clusters of orthologous groups. The functional classification was obtained from the KEEG database according to KEEG terms.

FIGURE S2 Analysis of membrane blebs cytotoxicity in splenocytes. Splenocytes were cultured with different concentrations of membrane blebs (1, 10, and 25 μg) to determine cytotoxicity. Cytotoxicity was determined indirectly through cell viability measurement by flow cytometry. Phytohemagglutinin (PHA) 20 μg, was used as positive control. The graphs showed the percentage of total live cells stimulated with membrane blebs compared with non-stimulated cells. *^∗∗∗^P* < 0.001.

FIGURE S3 Analysis of expression of surface markers on splenocytes from mice immunized with *B. abortus* membrane blebs. **(A)** Expression of the MHC-II molecule, CD11c and CD86 was evaluated with Mean intensity fluorescence (MFI) from gated APCs in the spleen of immunized mice with membrane blebs from *B. abortus* 2308 and RB51 strains. **(B)** Expression of the CD4^+^ and CD69 from gated T cells CD3^+^ was evaluated with Mean intensity fluorescence (MFI). **(C)** Also, the expression of the CD3 and CD68 from the cytotoxic T cells from gated CD3^+^CD4^–^ was measured in the splenocytes of immunized mice with blebs from *B. abortus* 2308 and RB51 strains. **(D)** The expression of the CD19 molecule and CD69 were evaluated with Mean intensity fluorescence (MFI) from gated CD19^+^ cells in the spleens of immunized mice with membrane blebs from *B. abortus* 2308 and RB51 strains. *^∗^P* < 0.05, *^∗∗^P* < 0.01, *^∗∗∗^P* < 0.001.

FIGURE S4 Detection of antigenic proteins in the *B. abortus* membrane blebs by Western Blot. The electrophoretic running was performed with 30 μg of proteins of blebs obtained from each strain. Then the gel was transferred to PVDF membrane, blocked and incubated with immunized mice serum. **(A)** Western blot incubated with serum from mice immunized with 2308 membrane blebs; lane 1, *B. abortus* 2308 membrane blebs; lane 2, *B. abortus* RB51 membrane blebs. **(B)** Western blot incubated with serum from mice immunized with *B. abortus* RB51 membrane blebs; lane 1, *B. abortus* 2308 membrane blebs; lane 2, *B. abortus* RB51 membrane blebs.

TABLE S1 Annotation of identified proteins of membrane blebs from *B. abortus* 2308 by LC-MS/MS.

TABLE S2 Annotation of identified proteins of membrane blebs from *B. abortus* RB51 by LC-MS/MS.
