# Supplementary figures and images for: Proteomic Analysis of Membrane Blebs of Brucella abortus 2308 and RB51 and Their Evaluation as an Acellular Vaccine
Source: Front Microbiol. 2019 Nov 29;10:2714. doi: 10.3389/fmicb.2019.02714 (PMC6895012; doi:10.3389/fmicb.2019.02714)

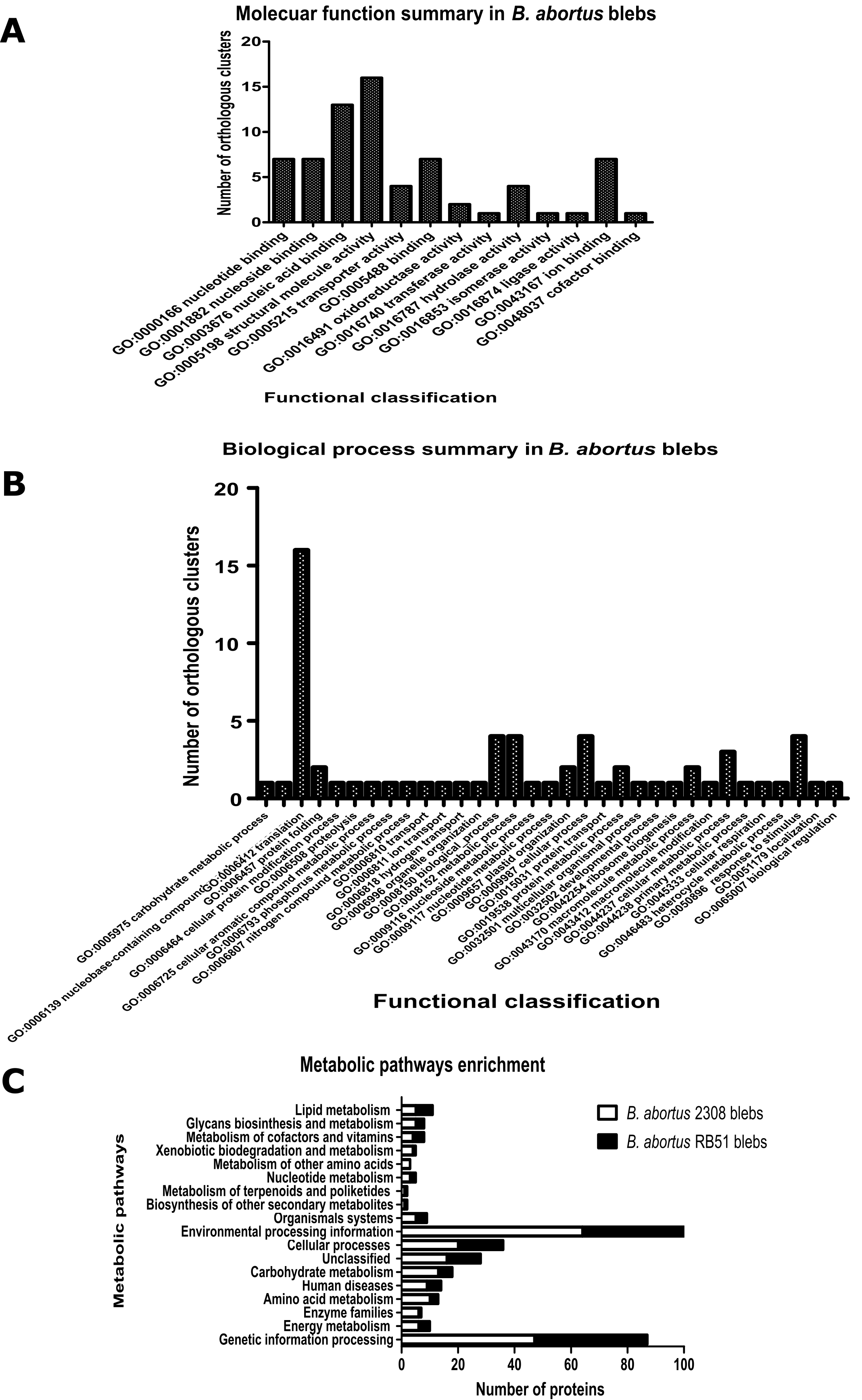

Supplement: FIGURE S1 — Enrichment of function for orthologous proteins identified in B. abortus membrane blebs. Distributions of B. abortus specific 112 orthologous protein sets in Molecular function (A) and biological process (B) GO slim terms. (C) Enrichment metabolic pathways of B. abortus 2308 and RB51 membrane blebs proteins. The proteins identified were sorted according to the indicated clusters of orthologous groups. The functional classification was obtained from the KEEG database according to KEEG terms. [file Image_1.tif]

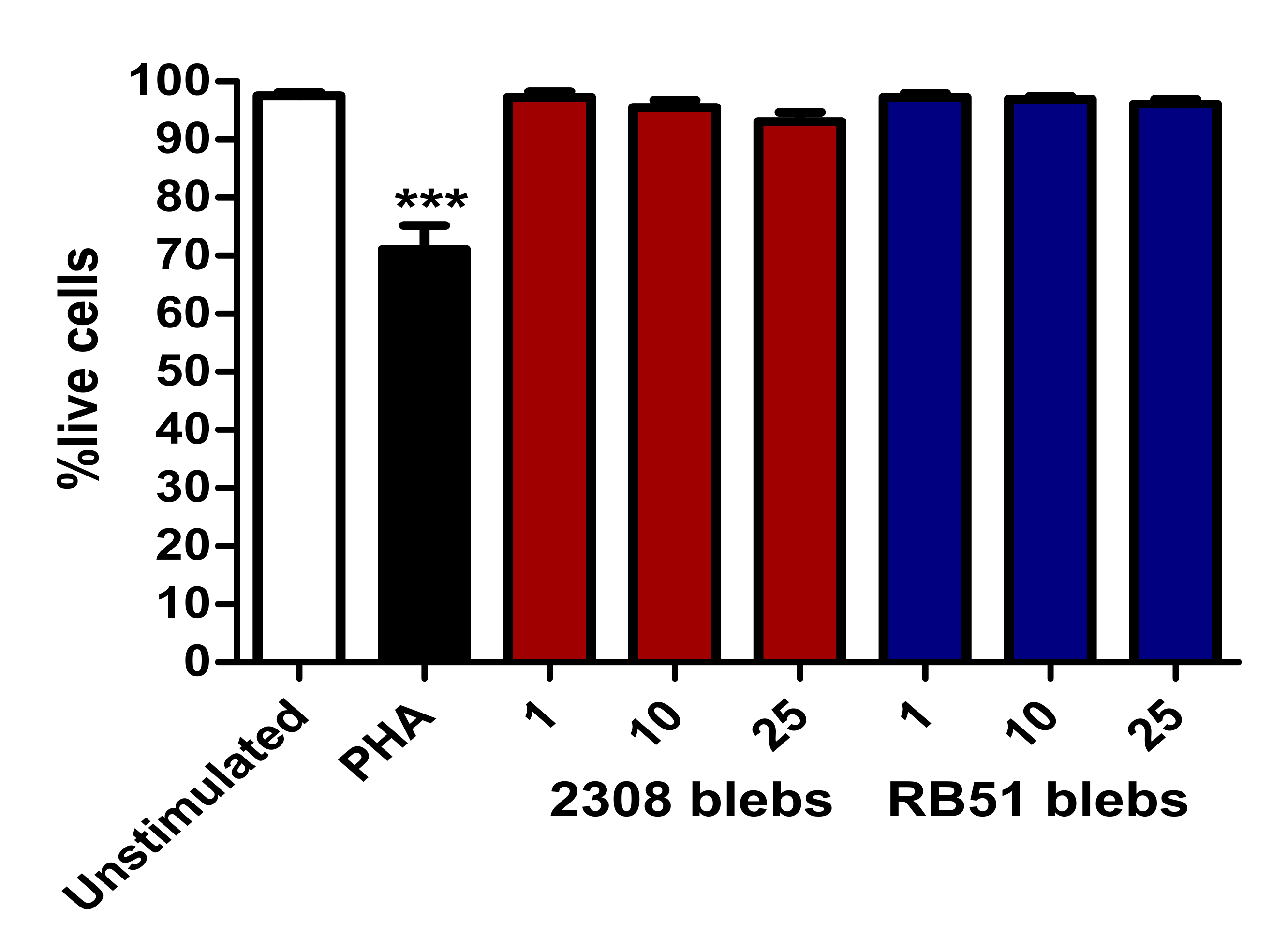

Supplement: FIGURE S2 — Analysis of membrane blebs cytotoxicity in splenocytes. Splenocytes were cultured with different concentrations of membrane blebs (1, 10, and 25 μg) to determine cytotoxicity. Cytotoxicity was determined indirectly through cell viability measurement by flow cytometry. Phytohemagglutinin (PHA) 20 μg, was used as positive control. The graphs showed the percentage of total live cells stimulated with membrane blebs compared with non-stimulated cells. ∗∗∗P < 0.001. [file Image_2.tif]

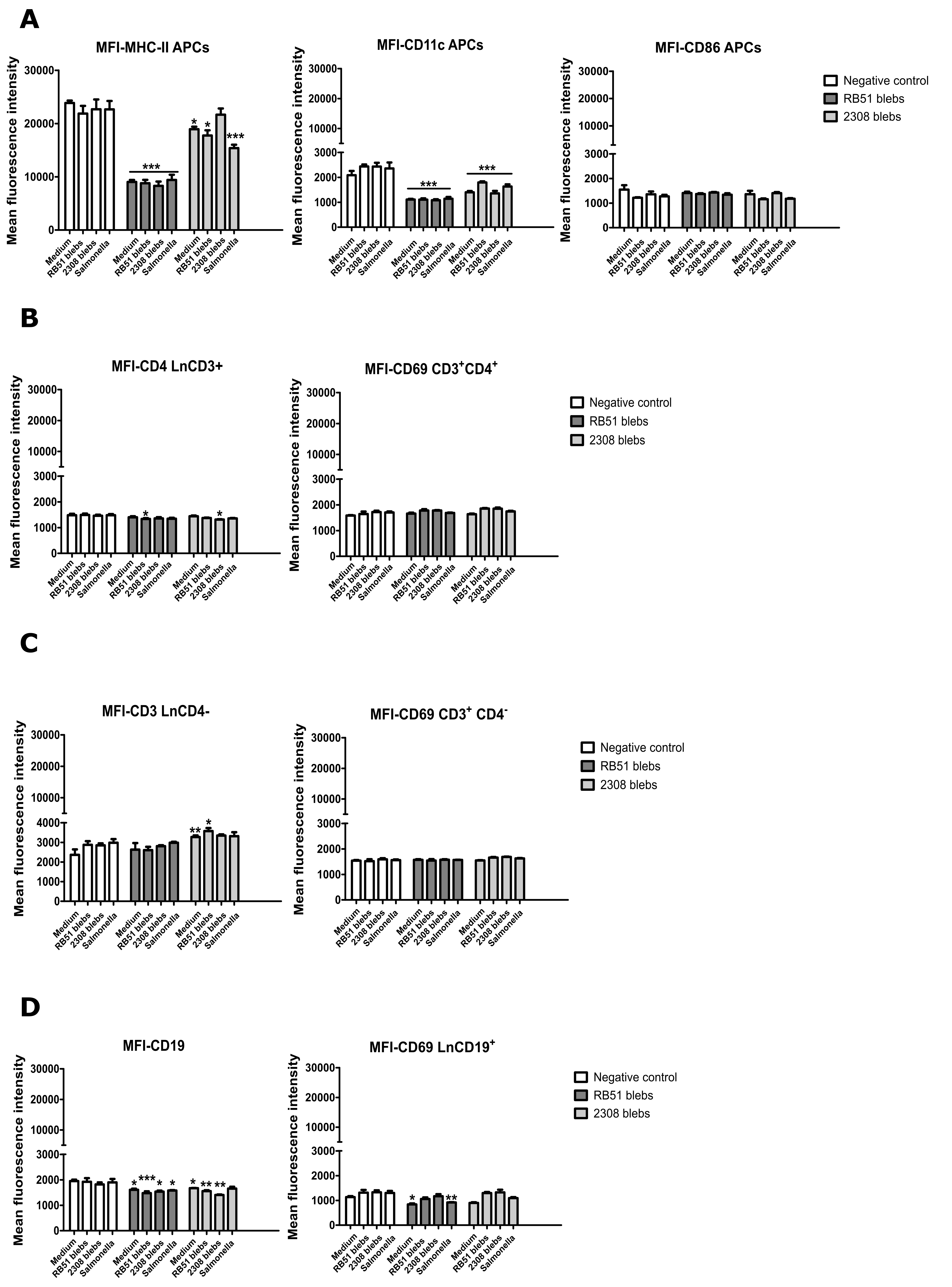

Supplement: FIGURE S3 — Analysis of expression of surface markers on splenocytes from mice immunized with B. abortus membrane blebs. (A) Expression of the MHC-II molecule, CD11c and CD86 was evaluated with Mean intensity fluorescence (MFI) from gated APCs in the spleen of immunized mice with membrane blebs from B. abortus 2308 and RB51 strains. (B) Expression of the CD4+ and CD69 from gated T cells CD3+ was evaluated with Mean intensity fluorescence (MFI). (C) Also, the expression of the CD3 and CD68 from the cytotoxic T cells from gated CD3+CD4– was measured in the splenocytes of immunized mice with blebs from B. abortus 2308 and RB51 strains. (D) The expression of the CD19 molecule and CD69 were evaluated with Mean intensity fluorescence (MFI) from gated CD19+ cells in the spleens of immunized mice with membrane blebs from B. abortus 2308 and RB51 strains. ∗P < 0.05, ∗∗P < 0.01, ∗∗∗P < 0.001. [file Image_3.tif]

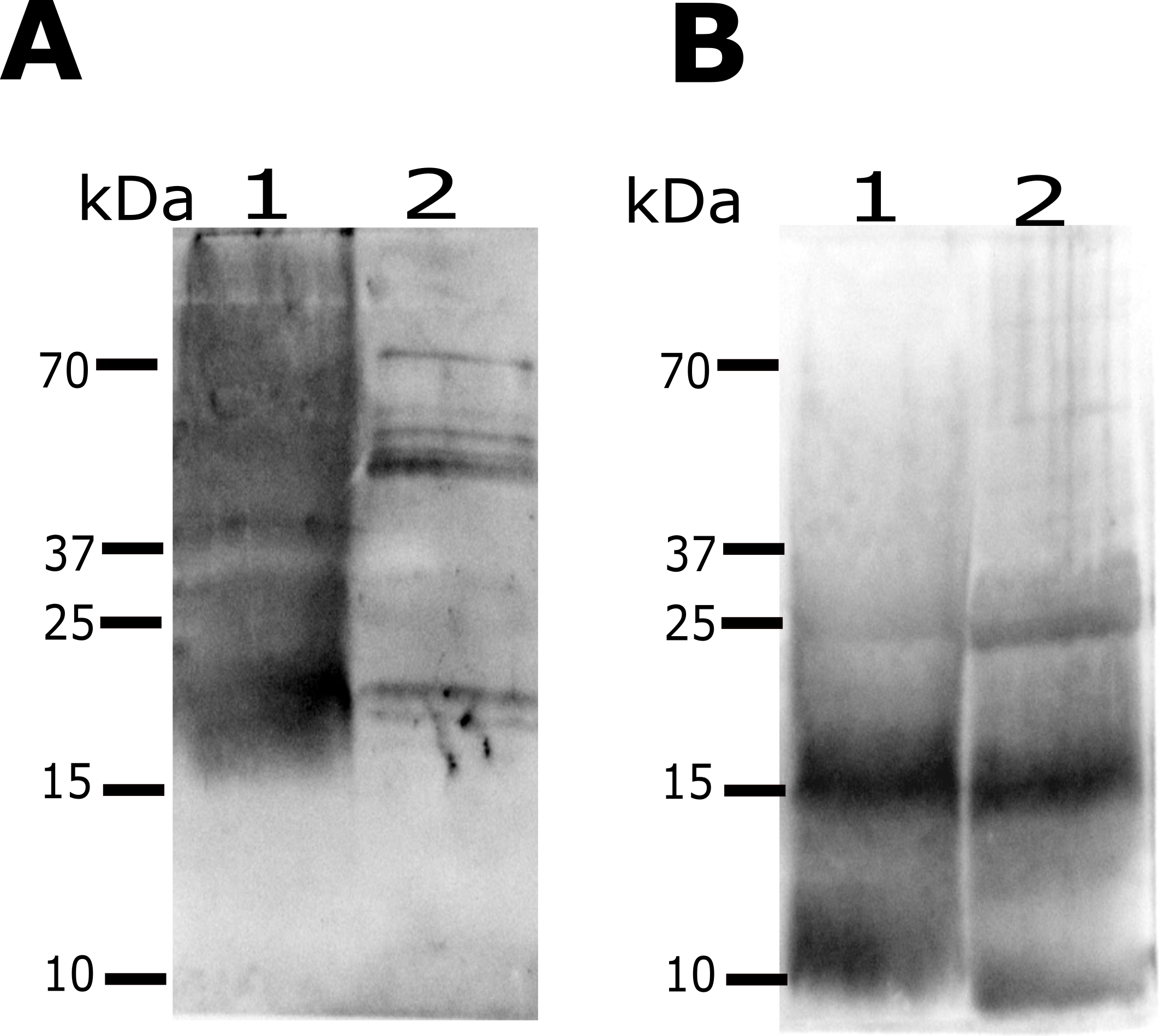

Supplement: FIGURE S4 — Detection of antigenic proteins in the B. abortus membrane blebs by Western Blot. The electrophoretic running was performed with 30 μg of proteins of blebs obtained from each strain. Then the gel was transferred to PVDF membrane, blocked and incubated with immunized mice serum. (A) Western blot incubated with serum from mice immunized with 2308 membrane blebs; lane 1, B. abortus 2308 membrane blebs; lane 2, B. abortus RB51 membrane blebs. (B) Western blot incubated with serum from mice immunized with B. abortus RB51 membrane blebs; lane 1, B. abortus 2308 membrane blebs; lane 2, B. abortus RB51 membrane blebs. [file Image_4.tif]
